# Electrochemical detection of single micelles through ‘nano-impacts’†
†Electronic supplementary information (ESI) available. See DOI: 10.1039/c5sc01635e



**DOI:** 10.1039/c5sc01635e

**Published:** 2015-06-18

**Authors:** H. S. Toh, R. G. Compton

**Affiliations:** a Department of Chemistry , Physical and Theoretical Chemistry Laboratory , Oxford University , South Parks Road , Oxford , OX1 3QZ , UK . Email: Richard.Compton@chem.ox.ac.uk

## Abstract

A new class of ‘soft’ particles, micelles, is detected electrochemically *via* ‘nano-impacts’ for the first time. Short, sharp bursts of current are used to indicate the electrical contact of a single CTAB (cetyltrimethylammonium bromide) micelle with an electrode *via* the oxidation of the bromide content. The variation in CTAB concentration for such ‘nano-impact’ experiments shows that a significant number of ‘spikes’ are observed above the CMC (critical micelle concentration) and this is attributed to the formation of micelles. A comparison with dynamic light scattering is also reported.

## Abstract 2

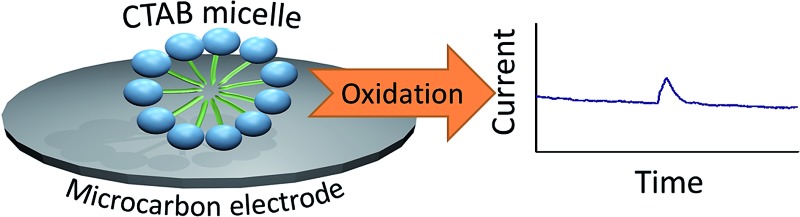
CTAB (cetyltrimethylammonium bromide) micelles are detected directly *via* the novel electrochemical method of ‘nano-impacts’ through oxidation of its bromide content.

## Introduction

‘Nano-impact’ chronoamperometry is a novel method developed to analyse single particles.^1^ It works by recording the electrochemical signal generated when a single particle hits the electrode held at a suitable potential.^2^ For direct electrochemical detection, a redox reaction of the particle occurs upon electrical contact with the electrode, Faradaic current is generated and this results in a short increase in current (‘spike’) on the chronoamperogram.

Typically, this is used to detect ‘hard’ metallic nanoparticles like silver,^2–4^ gold,^5,6^ nickel^4,6^ and mercury halides.^7,8^ However, ‘soft’ particles are also detectable through ‘nano-impacts’,^9–13^ starting with the work of Hasse *et al.* where lecithin liposomes were recorded through capacitative ‘spikes’.^9^ Recently, the direct oxidation of the encapsulated materials such as vitamin C^10^ and catecholamine hormones^11^ have been used to determine the presence of liposomes.

**Scheme 1:**
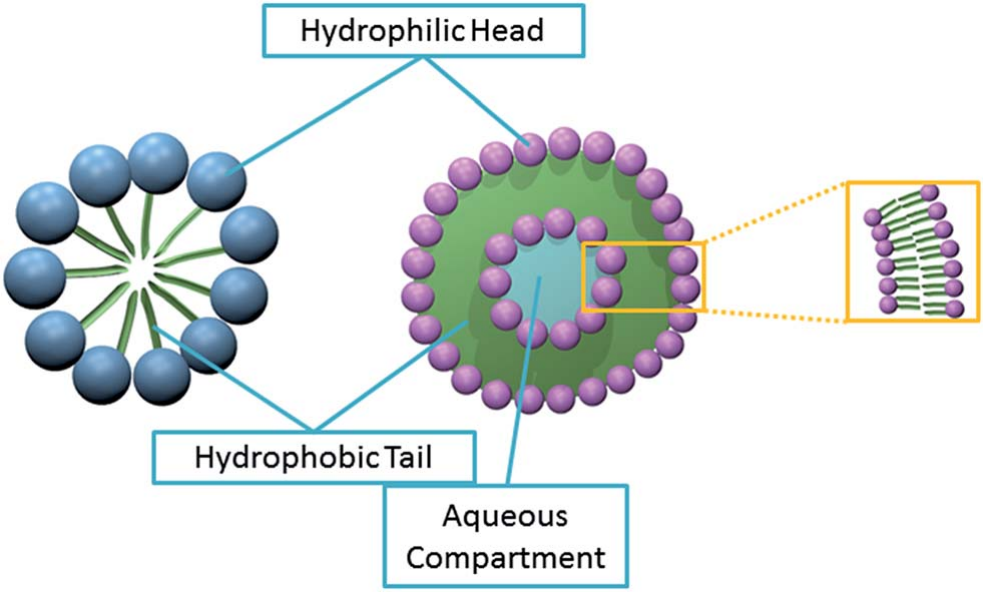
The structure of micelle (left) and liposome (right). The spheres represent the hydrophilic groups of the amphipathic molecule. The hydrophobic groups are represented by the green lines.

Hitherto, most ‘soft’ particles analysed by ‘nano-impacts’ are liposomes.^9–13^ These lipid vesicles are aqueous compartments enclosed by a lipid bilayer.^14^ Scheme 1 illustrates their capability to capture a small volume of aqueous solution. Therefore, detection is often based on the redox active components encapsulated within.^10–12^ However, in the current study, the direct detection of micelles using ‘nano-impacts’ is explored. These are globular structures with polar head groups surrounded by water whilst their hydrocarbon tails are isolated inside, facing one another and away from the aqueous environment.^14^ Scheme 1 shows the close packing of the hydrophobic groups of the micelle which confers thermodynamic stability.^15^ Within the digestive system, bile salts forms micelles to aid in the uptake of fat soluble vitamins (*i.e.* vitamin A, D, E and K).^16^ They are also often used as soap as they emulsify oil, allowing water to wash away oil-containing micelles.^17^

**Scheme 2:**
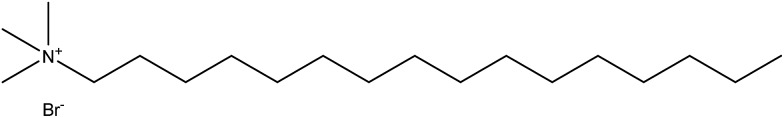
The chemical structure of cetyltrimethylammonium bromide (CTAB).

In the present study, cetyltrimethylammonium bromide (CTAB) is used as the analyte to form the micelles for detection. As shown in Scheme 2, it is a cationic agent containing a quaternary ammonium cation and a bromide anion.^18^ CTAB forms micelles as it only has a single hydrocarbon chain;^19^ molecules with two hydrocarbon tails prefer to form liposomes due to their bulky hydrophobic groups.^20^ In addition, CTAB is a regular reagent for DNA extraction in plants.^21–23^ Due to its importance, the critical micelle concentration (CMC) of this standard micellar agent is well-studied.^24–27^

Herein, the detection of CTAB micelles is performed through the electrochemical method of ‘nano-impacts’. The electrochemical oxidation of CTAB was first studied on a macro electrode system and compared to the oxidation of free bromide ions in aqueous solution. Next, ‘nano-impacts’ were used to determine the potential onset of the ‘spikes’ and the influence of CTAB concentration on the chronoamperograms. Dynamic light scattering was also performed to analyse the size distribution of the CTAB micelles.

## Results and discussion

First, a solution of CTAB was oxidised on a macro glassy carbon electrode *via* cyclic voltammetry to determine the oxidation potential arising from the bromide ion content. Next, the oxidation study of CTAB was performed on a carbon microdisc electrode to ensure that the data obtained on the two types of electrodes can be compared across both cyclic voltammetric and chronoamperometric data. Then, current–time transients were performed to observe the ‘spikes’ generated by the CTAB micelles impacting the microelectrode. The onset potential of the signals was determined by holding different potentials on the electrode during chronoamperometry. It is compared to the oxidation signal obtained in cyclic voltammetry to ensure the ‘spikes’ originated from the CTAB micelles. Next, the CTAB concentration was varied to determine its correlation to ‘spike’ frequency and magnitude. Last, dynamic light scattering was employed to determine the size distribution of the micelles and it is inferred that only the large CTAB micelles are detected *via* the ‘nano-impact’ method.

### Cyclic voltammetry studies

**Fig. 1:**
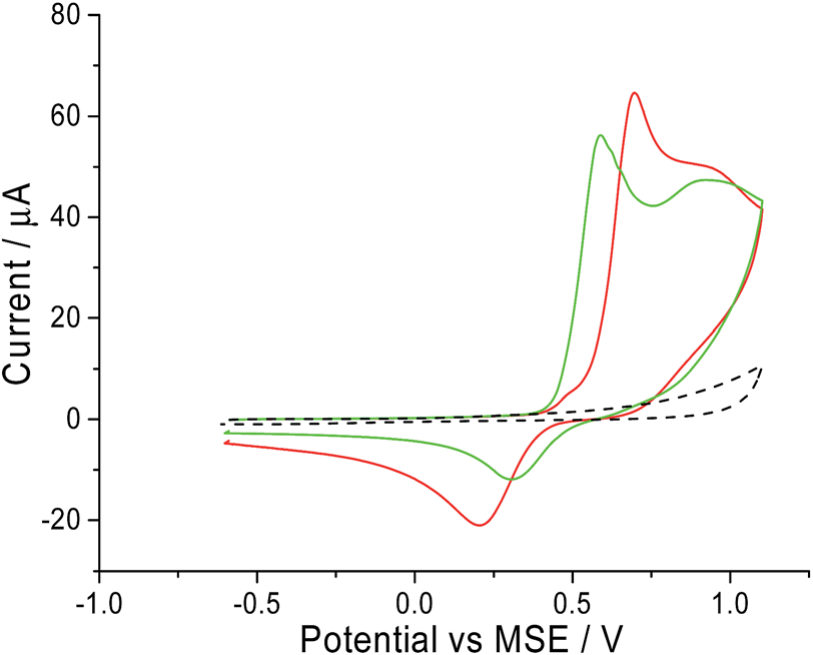
The cyclic voltammogram measured on a glassy carbon electrode in 0.10 M sodium nitrate at a scan rate of 25 mV s^–1^. Black dashed line: blank scan; red solid line: in electrolyte containing 5.0 mM CTAB; green solid line: in electrolyte containing 5.0 mM potassium bromide.

A freshly polished macro glassy carbon electrode was dipped into a solution containing 5.0 mM of CTAB and 0.10 M of sodium nitrate supporting electrolyte. A cyclic voltammetric scan started oxidatively from –0.6 V *vs.* MSE towards +1.1 V *vs.* MSE at a scan rate of 25 mV s^–1^. This experiment (red line in Fig. 1) gave two distinct peaks at +0.7 V and +0.9 V *vs.* MSE. In the absence of CTAB (black dashed line in Fig. 1), no oxidative signals are observed. To determine if the peaks arose from the bromide ion in CTAB, the same cyclic voltammetry experiment was performed with 5.0 mM of potassium bromide instead of CTAB. Two similar distinctive signals at +0.6 V and +0.9 V *vs.* MSE are recorded for potassium bromide (green line in Fig. 1). Overlaying the two voltammograms, the slight difference in oxidation potential can be attributed to the ion pairing present with CTAB. Thus, from literature, the two peaks correspond respectively to:^28,29^

1 1st wave: 3Br^–^ → Br–3 + 2*e*^–^

2 2nd wave: 2Br^–^ → Br_2_ + 2*e*^–^

Since these signals are both observed in the voltammogram of CTAB and potassium bromide, it is highly likely that the electrochemical oxidation of CTAB involves its bromide counter ion.

**Fig. 2:**
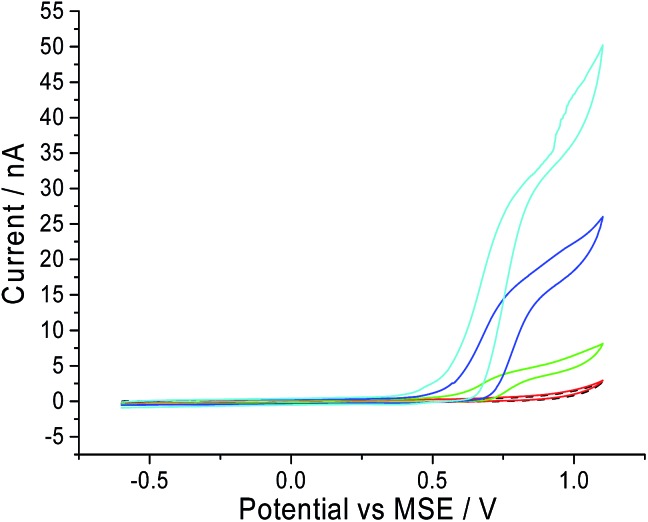
The cyclic voltammogram on a carbon microdisc electrode in 0.10 M sodium nitrate at a scan rate of 10 mV s^–1^. Black dashed line: blank scan; red solid line: 100 μM CTAB; green solid line: 1.0 mM CTAB; blue solid line: 5.0 mM CTAB; cyan solid line: 10.0 mM CTAB.

Next, the oxidation of CTAB on a carbon microdisc electrode was investigated to ensure that the mechanism remains similar to the one observed on a macro electrode. This is to provide a basis for chronoamperometric studies where a micro electrode was used to lower background noise. Thus, a carbon microdisc electrode was immersed in a solution of 0.10 M sodium nitrate electrolyte and various concentrations of CTAB. Cyclic voltammetry was performed with the same potential window of –0.6 V to +1.1 V *vs.* MSE at a scan rate of 10 mV s^–1^ and these voltammograms are summarised in Fig. 2. The increase in anodic current around +0.7 V *vs.* MSE is clearly noticeable at 1.0 mM, 5.0 mM and 10.0 mM of CTAB in Fig. 2 (green, blue and cyan line respectively). This corresponds to the first oxidation signal occurring on the macro glassy carbon electrode at +0.7 V *vs.* MSE in Fig. 1. In addition, at 10.0 mM CTAB, the voltammogram has a two-step increase in current at +0.7 V and +0.9 V *vs.* MSE which correlates to the two peaks (*i.e.* +0.7 V and +0.9 V *vs.* MSE) observed in Fig. 1. The slight difference in onset potential can be attributed to the common occurrence of finding reversible electrochemistry on a macro electrode appearing as less reversible on a microelectrode. Thus, there is no significant difference between the oxidation of CTAB on a macro glassy carbon electrode and a carbon microdisc electrode. At 100 μM of CTAB, no increase of anodic current is observed as the concentration has fallen below the detection limit of the cyclic voltammetric system.

### Chronoamperometric studies

The ‘nano-impact’ method involves performing current–time transients with a carbon microdisc electrode held at a fixed potential.^2,3^ As a single micelle comes into contact with the oxidising electrode surface, the redox species (*i.e.* CTAB) is oxidised, generating a ‘spike’.

Two different experiments were performed in this study to ensure that the signals are caused by CTAB micelles. First, multiple blank chronoamperometric scans were performed before the start of every ‘nano-impact’ experiment. The electrode was placed in 0.10 M sodium nitrate and held at a potential of +1 V *vs.* MSE for fifty seconds. No ‘spike’ was observed for blank scans in the absence of CTAB. ‘Spikes’ were only observed after an aliquot of CTAB is added into the solution.

**Fig. 3:**
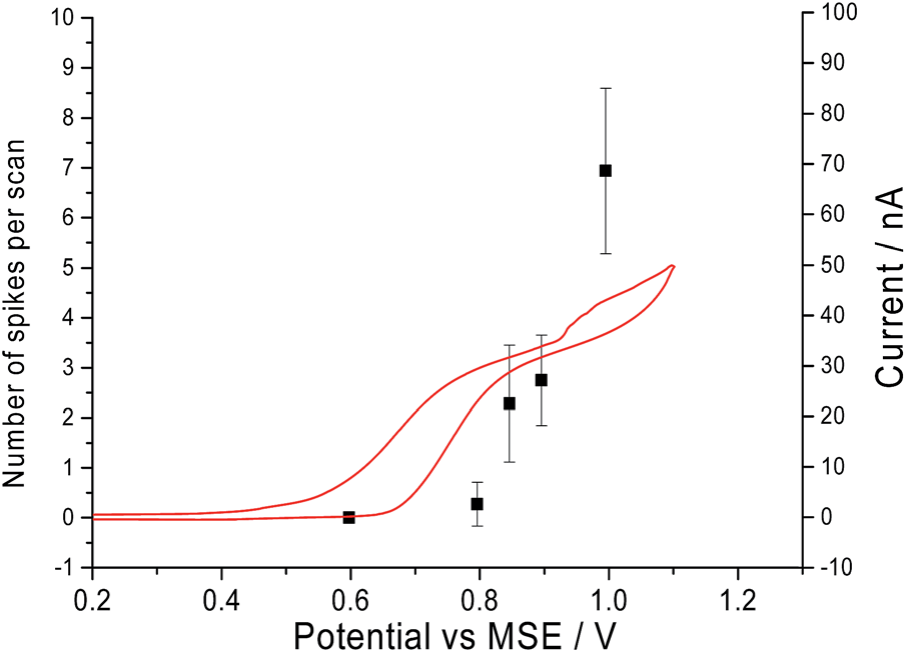
The cyclic voltammogram on a microcarbon electrode in 0.10 M sodium nitrate and 10.0 mM CTAB at a scan rate of 10 mV s^–1^ overlaid with a plot of the number of spikes observed per scan against the potential the chronoamperograms are performed.

Second, it was determined that the onset potential for the ‘spikes’ matches the oxidation potential observed in the cyclic voltammograms. Current–time transients were performed in a solution of 10.0 mM of CTAB and 0.10 M sodium nitrate at different potentials ranging from +0.6 V to +1 V *vs.* MSE. In Fig. 3, the number of ‘spikes’ observed per scan is overlaid with the cyclic voltammogram of a solution containing 10.0 mM CTAB and 0.10 M sodium nitrate. At +0.6 V *vs.* MSE, no ‘spike’ is seen in the chronoamperograms and no oxidation is occurring in cyclic voltammogram. At +0.8 V *vs.* MSE, a clear increase in anodic current is seen in the cyclic voltammogram and 3 ‘spikes’ are seen in a total of 11 current–time transients. At +1 V *vs.* MSE, a total of 229 ‘spikes’ are counted from 33 chronoamperograms whilst CTAB is oxidised in the cyclic voltammograms. Thus, the onset potential of the ‘spikes’ has a slight overpotential compared to its cyclic voltammogram counterpart. The small overpotential is required to oxidise the bromide content in the stabilised micelles compared to the non-micellar bromide ions in solution. Hence, the comparison of the onset potential of the ‘spikes’ and the oxidation signal in cyclic voltammogram indicated that the ‘spikes’ are caused by CTAB micelles. The mechanism of oxidation of the micelles might occur either *via* electron hopping as described by Amatore *et al.* for the case of a dendrimer molecule or *via* coupled oxidation of bromide ions and loss of cationic surfactant molecules.^30^

**Fig. 4:**
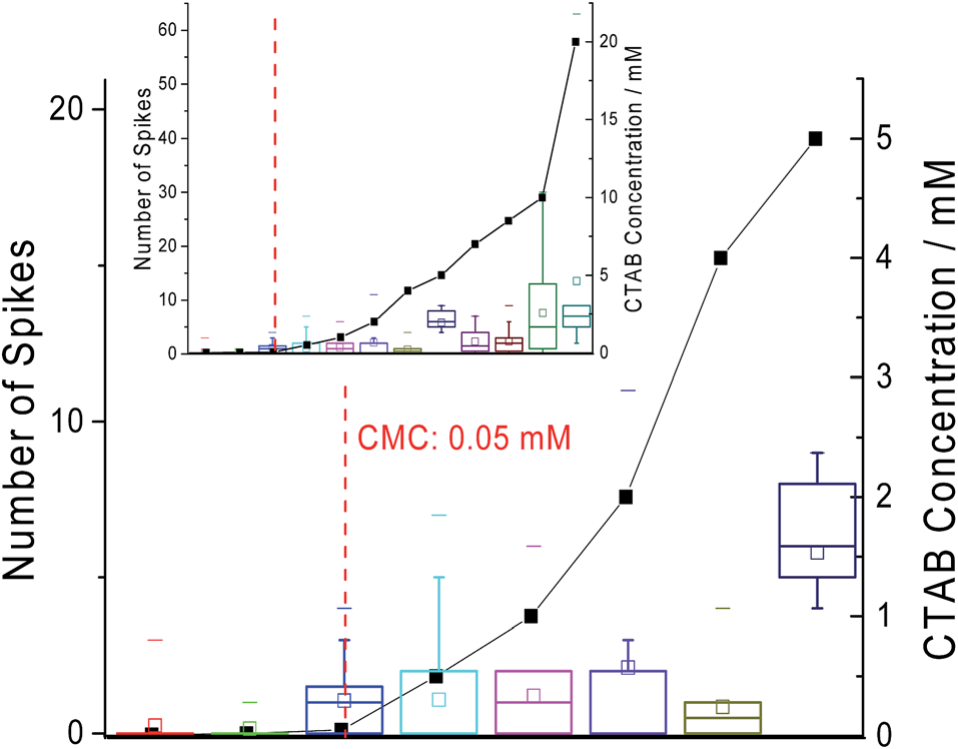
The close-up plot of the number of spikes observed per scan (box plot) and the concentration of CTAB at which the spikes are recorded (scatter plot). Inlay: the number of spikes seen per scan against the full range of CTAB concentration tested. Red dotted line: the critical micelle concentration of 0.05 mM from literature.^24^ The box reflects the 25th and 75th percentile. The short dash represents the maximum number of spikes observed per scan while the square represents the mean.

Next, chronoamperometric scans were performed across different concentrations of CTAB (*i.e.* 0.01 mM to 20 mM) in 0.10 M sodium nitrate to determine effects of concentrations on ‘spikes’. All fifty second current–time transients were recorded at +1 V *vs.* MSE with a carbon microdisc electrode. The individual chronoamperograms at each concentration can be found in Fig. S1 in the ESI.† Examples of the ‘spikes’ observed in the chronoamperograms are displayed in Fig. S2 of the ESI.† The background current increases with CTAB concentration due to the increase in free CTAB molecules present in the solution. In Fig. 4, the number of ‘spikes’ observed per scan are plotted against the CTAB concentration. It is seen that with increasing amount of CTAB, the number of ‘spikes’ observed increases. The onset of signals (at least one ‘spike’ per scan) coincides with the CTAB CMC of 0.05 mM.^24^ This indicates that the significant number of ‘spikes’ recorded are attributed to the CTAB micelles formed above CMC. This could possibly provide a novel method for CMC determination instead of the traditional technique of surface tension measurement.

**Fig. 5:**
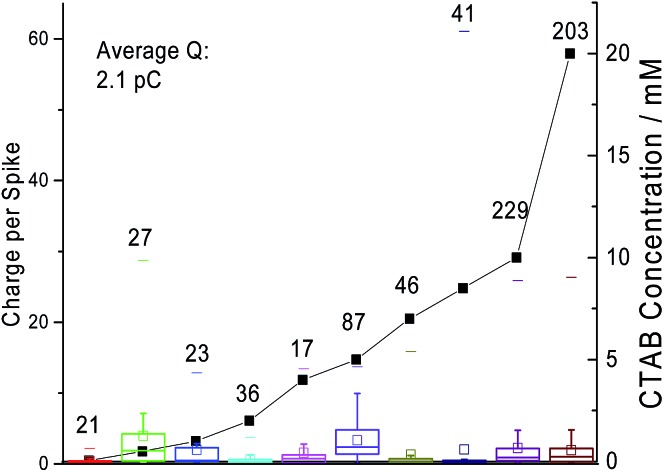
The charge under a spike (box plot) is plotted with the CTAB concentration where the spikes are recorded (scatter plot). The number of spikes recorded at each concentration is listed near the data point. The box reflects the 25th and 75th percentile. The short dash represents the maximum number of spikes observed per scan while the square represents the mean.

In Fig. 5, the average charge passed under a ‘spike’ is plotted against the amount of CTAB in solution with the standard deviations plotted as error bars. As observed in Fig. 5, there is no correlation between the amount of current passed and the CTAB concentration. The average charge measured per ‘spike’ across all concentrations of CTAB is 2.1 pC. If all the current measured is Faradaic, this corresponds to the oxidation of 1.3 × 10^7^ CTAB molecules given that CTAB oxidation is a one electron reaction. Thus, very large micelles must be responsible for the Faradaic charge measured. Additionally, there might be capacitive coupling whereby the Faradaic signal is amplified by the change in interfacial capacitance on impact. The possibility of ‘spikes’ originating only from a capacitative nature is ruled out because a control experiment was performed at negative potentials (*i.e.* –0.8 V to –1 V *vs.* MSE) and no ‘spikes’ were recorded.

**Fig. 6:**
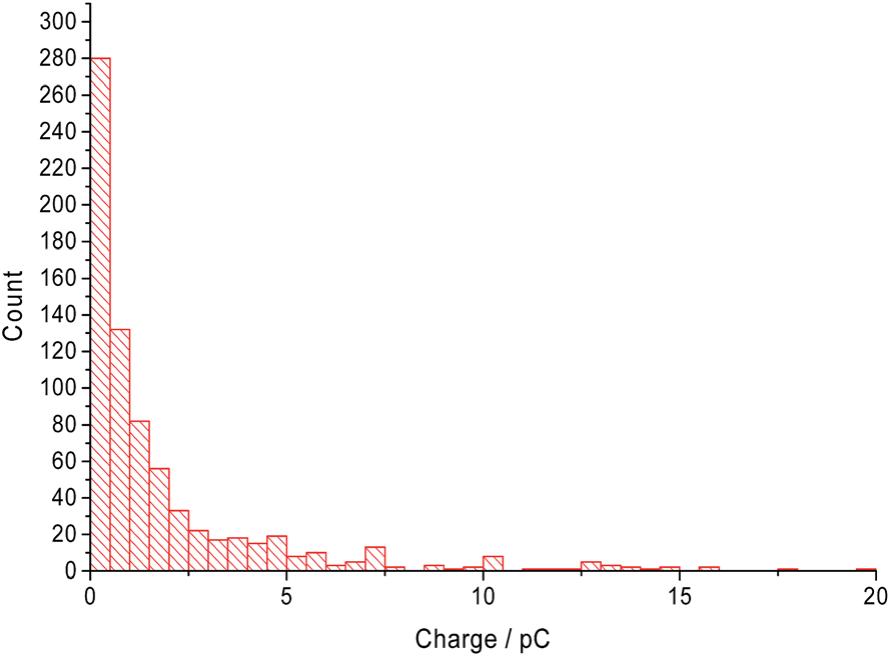
The charge distribution of the current recorded under a spike.

The distribution of the charge passed under each ‘spike’ across all CTAB concentrations are displayed in Fig. 6. Most of the recorded ‘spikes’ are small and 37% of them have a charge lower than 0.5 pC. In fact, 80% of all ‘spikes’ recorded contained less than 3 pC. From Fig. 6, the charge distribution resembles the tail of a log normal distribution. Thus, it is hypothesized that only the large CTAB micelles are recorded on the chronoamperograms under the assumption that the micelles follow a log normal size distribution. This hypothesis is supported by the data in Fig. 4 and 5. In Fig. 4, as the concentration of CTAB increases, the number of large micelles grows, thus leading to an increase in frequency of ‘spikes’. However, the average charge per ‘spike’ is independent of CTAB concentration (Fig. 5). This is because size differences among the larger micelles do not change the current measured significantly compared to micelles of a smaller size. Assuming a surface reaction, a 2% increase in current is observed when a particle changes from 100 nm to 101 nm while a 21% increase is seen as a particle changes from 10 nm to 11 nm. Therefore, to prove that the CTAB micelles follow a log normal size distribution, dynamic light scattering was next performed to support the hypothesis.

### Dynamic light scattering studies

**Fig. 7:**
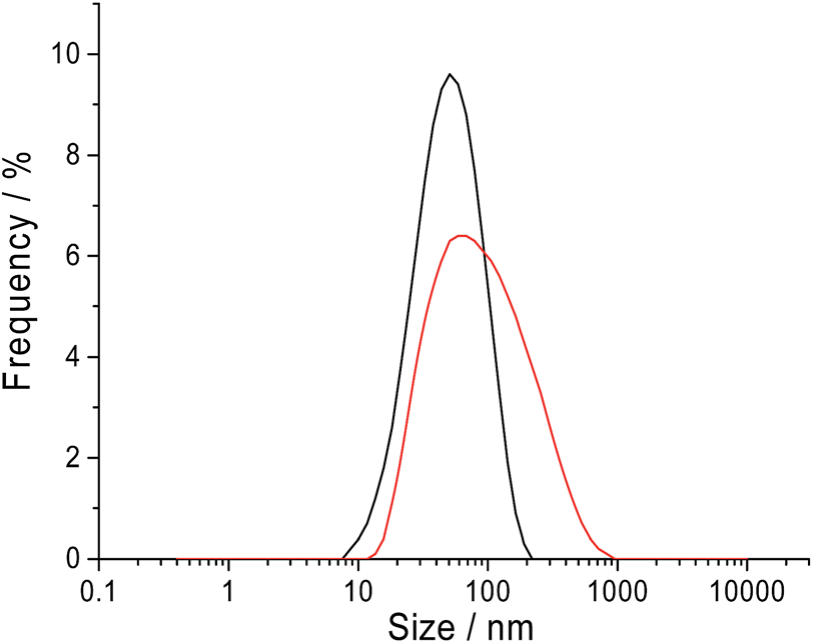
The intensity weighted hydrodynamic diameter of the CTAB micelles in sodium nitrate solution containing 10.0 mM CTAB at 25 °C. Black: 0.10 M sodium nitrate; red: 0.50 M sodium nitrate.

Dynamic light scattering was performed on a sample of 10.0 mM CTAB in 0.10 M sodium nitrate to measure the size distribution of the CTAB micelles. The intensity weighted size distribution is summarised in Fig. 7. The black line shows the CTAB micelle sample following a log normal distribution with an average hydrodynamic diameter of 41.2 nm and a mode of 56.7 nm. The sample has a polydispersity index of 0.245. This observation of a log normal size distribution strongly supports the hypothesis of ‘nano-impact’ method measuring only the larger micelles and the tail of the size distribution is detected through the chronoamperograms in Fig. 6.

To carry this hypothesis further, the electrolyte concentration was varied for the dynamic light scattering and ‘nano-impact’ experiments. From literature, it is known that smaller micelles are formed in the presence of a lower electrolyte concentration.^24^ From Table 1, it is observed that at 0.05 M sodium nitrate, the CTAB micelles has an average diameter of 11.7 nm compared to 41.2 nm (0.10 M sodium nitrate, black line in Fig. 7) and 61.7 nm (0.50 M sodium nitrate, red line in Fig. 7) at higher concentrations of electrolyte. The counterpart electrochemical control experiment was performed with 10.0 mM CTAB in a lower electrolyte concentration of 0.05 M sodium nitrate *via* ‘nano-impact’. No ‘spike’ was observed in the chronoamperograms. This is likely due to the absence of very large micelles in the lower electrolyte concentration environment. Thus, this further strengthens the hypothesis where ‘nano-impacts’ are detecting the large micelles present in the CTAB solution. It is to be taken note that dynamic light scattering was attempted with 0.05 M sodium nitrate in the current study. However, the polydispersity nature of the sample resulted in an unsuccessful measurement.

**Table 1:** Dynamic light scattering results of the solutions containing 10.0 mM CTAB and various concentrations of sodium nitrate

[NaNO_3_]/M	Average hydrodynamic diameter/nm	Mode/nm	Polydispersity index	Temperature/°C	Ref.
0.05	11.7	—	0.23	30	24
0.10	41.2	56.7	0.24	25	Current study
	38.4	—	0.24	30	24
0.50	61.7	119.5	0.43	25	Current study
	71.0	—	0.51	30	24

Despite the micelles having an average hydrodynamic diameter of 41.2 nm at 0.10 M sodium nitrate, most of smaller particles are not detected *via* ‘nano-impacts’. In the literature, silver nanoparticles of 6 nm diameter have been successfully analysed and sized.^8^ Comparing with a previous study where the exact same set-up and microcarbon electrode were utilised, silver nanoparticles of an average diameter of 24 nm were detected.^3^ The silver nanoparticles gave an average charge of 0.66 pC with a background noise level of 6 pA. In the current study, the average charge measured was 2.1 pC. As mentioned previously, the background current increases with the CTAB concentration in Fig. S1.† It is also observed that the variation of CTAB concentration resulted in different background noise of 6 pA (0.5 mM CTAB), 20 pA (4.0 mM CTAB) and 40 pA (20.0 mM CTAB). Thus, the magnitude of the background noise correlated directly with the amount of CTAB present in the solution. With a higher noise, there is a greater difficulty to differentiate the signals from the background noise. Hence, the rest of the smaller micelles remain undetected as the ‘spikes’ they generate cannot be resolved from the background noise. Therefore, ‘nano-impacts’ has detected the larger single CTAB micelles towards the tail of the size distribution.

## Experimental

### Chemicals

Cetyltrimethylammonium bromide (95%, ((C_16_H_33_)N(CH_3_)_3_Br)) and ethanol (≥99.8%, C_2_H_5_OH) was purchased from Sigma-Aldrich, Dorset, UK. Sodium nitrate (>99.5%, NaNO_3_) was supplied from Fisons Scientific Equipment, Loughborough, UK. Ultrapure water from Millipore with resistivity no less than 18.2 MΩ cm at 25 °C was used to prepare all solutions.

### Electrochemical apparatus

A μAutolab II (Metrohm-Autolab BV, Utrecht, The Netherlands) was used to control the electrochemical experiments with the software of NOVA 1.10. All electrochemistry experiments were performed in a Faraday cage with a three electrode system. For cyclic voltammetry experiments, a glassy carbon electrode (CH instruments, Austin, USA) of 3.0 mm diameter was used. It was polished on diamond spray (Kemet, Kent, UK) in the size sequence of 3.0 μm, 1.0 μm and 0.1 μm to a mirror finish. For chronoamperometric experiments, a carbon microdisc working electrode (BASi, West Lafayette, USA) of radius 4.9 μm was used. It was polished on alumina powder (Buehler, Coventry, UK) in the size sequence of 1.0 μm, 0.3 μm and 0.05 μm before experiments. The reference electrode was a standard MSE (mercury/mercurous sulphate reference electrode [Hg/Hg_2_SO_4_, K_2_SO_4_ (saturated)], +0.62 V *vs.* standard hydrogen electrode) (BASi, West Lafayette, USA).^31^ The counter electrode was a platinum mesh (99.99%) (Goodfellow Cambridge Ltd, Huntingdon, UK). All electrochemical measurements were thermostated at 25 ± 1 °C.

### Chronoamperometric experiments

Prior to every chronoamperometric experiments, the electrochemical cell was cleaned by sonication in a mixture of ethanol and water (1 : 1 ratio) for at least 30 minutes to avoid any contamination by leftover CTAB. All electrodes were rinsed with ethanol and ultrapure water to ensure no CTAB is carried over from previous experiments. Fifty seconds long chronoamperometric scans with a sampling time of 0.0005 seconds were recorded. The number and magnitude of the ‘spikes’ were determined by the software of SignalCounter. The software SignalCounter was developed by Dr Dario Omanović from Division for Marine and Environmental Research, Ruđer Bošković Institutue, Zagreb, Croatia for in-house use as a part of a collaboration.^32,33^ This software is programmed to pick up ‘spikes’ of a minimum intensity of 5 pA height. A linear baseline was taken and the charge underneath the peak calculated. The baseline was taken at the midpoint of the average noise to minimize the amount of background taken as signal. All signals were further checked manually to differentiate actual ‘spikes’ from noise through the signal shape.

### Dynamic light scattering

Dynamic light scattering (Zetasizer NanoZS, Malvern Instruments Ltd, Malvern, UK) was used as a technique to characterise the CTAB micelles in the solution at 25 °C. A red laser of 633 nm was used to determine the size of the micelles in 10 mM CTAB solution with various concentration of electrolyte.

## Conclusions

This work reports for the first time, ‘nano-impacts’ to be a novel method for the detection of large CTAB micelles. From the cyclic voltammetric experiments, it is found that CTAB oxidation is attributed to the oxidation of its bromide ion. This one electron oxidation is responsible for the Faradaic current to generate ‘spikes’ in ‘nano-impact’. In the chronoamperometric scans, the onset potential of the ‘spikes’ matches the oxidation potential of CTAB in the cyclic voltammogram. Hence, the signals are attributed to the CTAB micelles present in the solution. By varying the concentration of CTAB, it is found that the number of ‘spikes’ per scan increases with concentration as more CTAB micelles are formed. Comparing the charge distribution of the ‘spikes’ and dynamic light scattering data, it is concluded that large CTAB micelles are detectable by ‘nano-impacts’. This represents an entirely new class of ‘soft’ particles that can be studied *via* this means.
