# Supplementary material for: Electrochemical detection of single micelles through ‘nano-impacts’
Source: Chem Sci. 2015 Jun 18;6(8):5053–8. doi: 10.1039/c5sc01635e (PMC5664171; doi:10.1039/c5sc01635e)
Supplement: Supplementary file 1 [file SC-006-C5SC01635E-s001.pdf]

Electronic Supplementary Material (ESI) for Chemical Science.
This journal is © The Royal Society of Chemistry 2015

Supporting Information for **Electrochemical Detection of Single Micelles through 'Nano-Impacts'**

By Her Shuang Toh[1], and Richard G. Compton[1]*

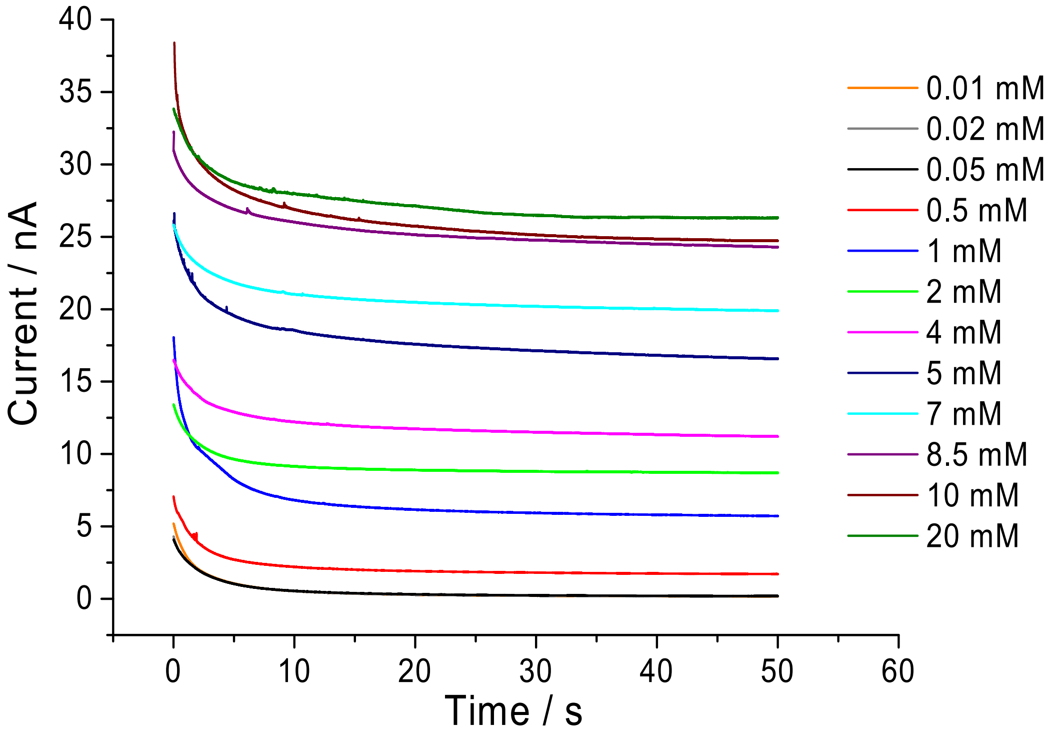

Figure S1. The chronoamperograms performed on solutions with 0.1 M sodium nitrate and different CTAB concentrations.

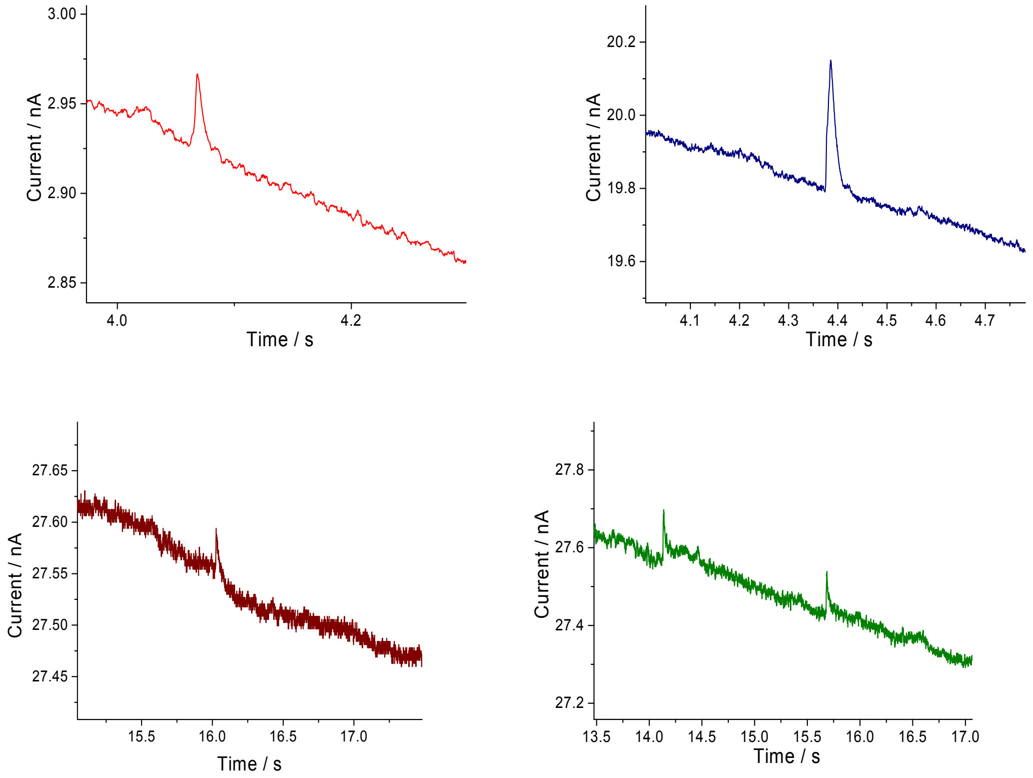

Figure S2. Examples of 'spikes' observed in the chronoamperogram at CTAB concentrations of A) 0.5 mM B) 5 mM C) 10 mM D) 20 mM.
